# Interleukin-10–mediated regenerative postnatal tissue repair is dependent on regulation of hyaluronan metabolism *via* fibroblast-specific STAT3 signaling

**DOI:** 10.1096/fj.201600856R

**Published:** 2016-11-30

**Authors:** Swathi Balaji, Xinyi Wang, Alice King, Louis D. Le, Sukanta S. Bhattacharya, Chad M. Moles, Manish J. Butte, Vinicio A. de Jesus Perez, Kenneth W. Liechty, Thomas N. Wight, Timothy M. Crombleholme, Paul L. Bollyky, Sundeep G. Keswani

**Affiliations:** * Laboratory for Regenerative Tissue Repair, Division of Pediatric Surgery, Texas Children’s Hospital, Baylor College of Medicine, Houston, Texas, USA;; † Division of Immunology, Department of Pediatrics, Stanford University, Stanford, California, USA;; ‡ Division of Pulmonary and Critical Care Medicine, Department of Medicine, Stanford University, Stanford, California, USA;; § Center for Children’s Surgery, Children’s Hospital Colorado, University of Colorado School of Medicine, Aurora, Colorado, USA;; ¶ Matrix Biology Program, Benaroya Research Institute, Seattle, Washington, USA; and; ‖ Division of Infectious Diseases, Department of Medicine, Stanford University, Stanford, California, USA

**Keywords:** fibrosis, scarless, wound healing, extracellular matrix, inflammatory cytokines

## Abstract

The cytokine IL-10 has potent antifibrotic effects in models of adult fibrosis, but the mechanisms of action are unclear. Here, we report a novel finding that IL-10 triggers a signal transducer and activator of transcription 3 (STAT3)–dependent signaling pathway that regulates hyaluronan (HA) metabolism and drives adult fibroblasts to synthesize an HA-rich pericellular matrix, which mimics the fetal regenerative wound healing phenotype with reduced fibrosis. By using cre-lox–mediated novel, inducible, fibroblast-, keratinocyte-, and wound-specific STAT3-knockdown postnatal mice—plus syngeneic fibroblast cell-transplant models—we demonstrate that the regenerative effects of IL-10 in postnatal wounds are dependent on HA synthesis and fibroblast-specific STAT3-dependent signaling. The importance of IL-10–induced HA synthesis for regenerative wound healing is demonstrated by inhibition of HA synthesis in a murine wound model by administering 4-methylumbelliferone. Although IL-10 and STAT3 signaling were intact, the antifibrotic repair phenotype that is induced by IL-10 overexpression was abrogated in this model. Our data show a novel role for IL-10 beyond its accepted immune-regulatory mechanism. The opportunity for IL-10 to regulate a fibroblast-specific formation of a regenerative, HA-rich wound extracellular matrix may lead to the development of innovative therapies to attenuate postnatal fibrosis in organ systems or diseases in which dysregulated inflammation and HA intersect.—Balaji, S., Wang, X., King, A., Le, L. D., Bhattacharya, S. S., Moles, C. M., Butte, M. J., de Jesus Perez, V. A., Liechty, K. W., Wight, T. N., Crombleholme, T. M., Bollyky, P. L., Keswani, S. G. Interleukin-10–mediated regenerative postnatal tissue repair is dependent on regulation of hyaluronan metabolism *via* fibroblast-specific STAT3 signaling.

Tissue fibrosis is the end phenotype of several diseases that result in significant morbidity and mortality (1). The common theme of all of these pathologies, such as myocardial infarction, dermal scarring, and renal and pulmonary fibrosis, is a fibroblast-mediated, altered collagen-rich extracellular matrix (ECM) (2). Fetal tissues have a striking ability to heal cutaneous wounds scarlessly (3, 4). This ability is lost in late gestation and is replaced with a scarring phenotype that is similar to the default healing nature of all postnatal repairs (5). As such, fetal dermal wound healing is a naturally occurring model of attenuated postinjury fibrosis that may serve as a blueprint for developing antifibrotic therapies (6). Fetal regenerative phenotype has distinct characteristics, including an attenuated inflammatory response and fetal fibroblasts (FFBs) with unique characteristics that are different from those of adult fibroblasts (AFBs) (7, 8). These FFBs generate an ECM that is characterized by persistent increase in the levels of hyaluronan (HA) compared with those generated by AFBs (9, 10).

HA is a large glycosaminoglycan that consists of a linear chain of repeating disaccharides. Virtually all organs generate some HA, but half of the total HA in the body is in the skin (11, 12). Synthesis of HA involves 3 HA synthases (HAS1, HAS2, and HAS3), which assemble UDP-glucuronic acid and UDP-N-glucosamine at the plasma membrane, forming a glycosaminoglycan chain. HAS1 and HAS2 are associated with high-molecular-weight HA (HMW-HA), which is noninflammatory in nature, whereas HAS3 produces low-molecular-weight HA, which has relatively greater proinflammatory effects (13). Dermal wounds created in mice that are deficient in both HAS1 and HAS3 showed increased inflammation and earlier myofibroblast differentiation, which resulted in increased fibrosis compared with control mice (14). Conversely, overexpression of HAS1 or application of HMW-HA has been shown to reduce fibrosis in dermal wound models (15, 16). Degradation of HA is regulated by hyaluronidase (HYAL)-related genes. HYAL1 and HYAL2 are known to have significant roles in degrading HA as a result of their expression pattern in skin (17–20). HYAL2 hydrolyzes HMW-HA (21). In addition, KIAA1199, a relatively new gene with unknown function, but that we know is related to deafness, has been shown to play a role in HA binding and depolymerization and is expressed predominantly by dermal fibroblasts in normal skin (22).

Fibroblast is the main source of HA in the skin and is also the cell that is the primary arbiter of scar formation in postnatal wounds (9, 10). FFBs are highly migratory, which may contribute to the fetal regenerative phenotype (23). It has been shown that the migratory capability of FFBs is dependent on their ability to generate pericellular matrices (PCMs) that are IL-10 dependent and HA rich (24). Cumulative past data suggest that, compared with other components that characterize the ECM, HA-rich matrices may provide a conducive framework for FFBs to traverse the wound bed more efficiently, leaving less collagen deposition and, thus, less scar formation (25–29). Consistent with this model, the migratory phenotype of FFBs can be recapitulated in AFBs by administration of IL-10; however, it was dependent on HA induced by IL-10 (24).

IL-10 is a potent anti-inflammatory cytokine (30) that prevents fibrosis in several models, including dermal wounds (31–33), myocardial infarction (34), and both lung (35) and kidney injury (36). Several lines of evidence demonstrate that IL-10 plays an essential role in the fetal regenerative phenotype. Levels of IL-10 are elevated in midgestation fetal skin that heals without scar formation (32), but fetal skin wounds from mice that are deficient in IL-10 at a similar gestational age form scars (37). Most convincingly, overexpression of IL-10 in adult skin induces wounds to heal without scar (31, 32, 38). Most explanations of the regenerative effects of IL-10 invoke its known anti-inflammatory properties and effects on local leukocytes and other inflammatory cells (39).

It has been recently suggested in fetal wounds that IL-10 has an essential role in the synthesis of an HA-rich ECM, which is essential for their scarless wound repair (40). Signal transducer and activator of transcription 3 (STAT3) has also been shown to play an essential role in the fetal regenerative phenotype. STAT3 inhibition in fetal wounds attenuates the formation of the characteristic HA-rich wound ECM and impairs the ability to re-epithelialize efficiently (41). However, it is unknown whether these key signaling factors that drive the scarless healing phenotype in the fetus are also present in the AFBs and postnatal wounds. Molecular mechanisms that underlie IL-10–mediated HA synthesis by AFBs and their potential contributions to postnatal regenerative wound repair have not been examined. This information is vital to the development of IL-10 as an antifibrotic agent in adult wounds.

Here, we have asked whether IL-10 regulates levels of HA in postnatal tissue. In particular, we have tested the hypothesis that IL-10 promotes generation of an HA-rich ECM by AFBs *via* a STAT3-dependent mechanism that results in postnatal regenerative wound healing. To test this hypothesis, we performed gain- and loss-of-function experiments in AFBs *in vitro*, and complemented these studies with *in vivo* experiments in an excisional murine wound model and several sets of novel transgenic mice.

## MATERIALS AND METHODS

### Primary cell isolation and culture

Primary fetal (E14.5) and adult (8–10 wk) murine dermal fibroblasts were isolated from the skin of C57BL/6J mice (The Jackson Laboratory, Bar Harbor, ME, USA) by using standard isolation protocols (42). Fibroblast cultures were routinely maintained at 37°C under 5% CO_2_ in a humidified chamber and cultured in DMEM (Thermo Fisher Scientific, Waltham, MA, USA) that was supplemented with 10% bovine growth serum (BGS; Hyclone, Logan, UT, USA), 100 U penicillin, 100 µg streptomycin, and 0.25 µg amphotericin B (Thermo Fisher Scientific). All cells used were between passages 5 and 10.

For all experimental procedures, cells were subcultured and allowed to settle overnight in culture medium that contained 10% BGS. Cells were then serum starved in culture medium with 2% BGS. After 24 h of serum starvation, various treatments were added to the conditioned medium (*n* = 3 per time point), and cells from 2 different passages were included.

### Particle exclusion assay

Exclusion of fixed red blood cells was used to visualize fibroblast pericellular coats (40). Fibroblasts were plated at a density of 7.5 × 10^4^ cells/well in 6-well plates (BD Falcon, Bedford, MA, USA). We studied the effects of the addition of murine IL-10 (200 ng/ml; Peprotech, Rocky Hill, NJ, USA), and 4-methylumbelliferone (4-MU), an HA synthase inhibitor (0.3 mM; Sigma-Aldrich, St. Louis, MO, USA). HA composition of the PCM was analyzed by enzymatic digestion of HA with 1 U/ml of HYAL from *Streptomyces* *hyalurolyticus* (H1136; Sigma-Aldrich), which was added to the culture medium for 20 min at the conclusion of the 24-h treatment time. Cells were rinsed in PBS, and 500 μl glutaraldehyde-stabilized sheep erythrocytes (1 × 10^8^ cells/ml; Intercell Technologies, Jupiter, FL, USA) were added to each well and allowed to settle for 10 min. Individual cells were imaged at ×20 magnification with an Axiovert 100M inverted phase contrast microscope (Carl Zeiss, Thornwood, NY, USA), which showed the outline of the cell body and the outline of the HA coat as a halo around the cells bounded by the red blood cells.

### Computer-assisted morphometric analysis of the PCM

Photomicrographs of randomly selected cells (20–30 cells/treatment condition) were obtained at the same magnification (×20). PCM ratio was calculated as the ratio of total PCM area to area of the cell body (40).

### Scanning electron microscopy of the PCM

Cells were washed and fixed in Karnovsky’s fixative composed of 2% (v/v) paraformaldehyde, 2.5% (v/v) glutaraldehyde, 3 mM calcium chloride, 5% (w/v) sucrose, and 0.2% (w/v) ruthenium red in cacodylate buffer (0.1 M) at pH 7.3 for 1 h at 22°C. Cells were rinsed in washing solution made of 3 mM calcium chloride, 5% (w/v) sucrose, and 0.1% (w/v) ruthenium red, and were postfixed in solution that contained 1% (w/v) osmium tetroxide and 0.05% (w/v) ruthenium red for 1 h at 22°C (Sigma-Aldrich). Cells were rinsed several times in PBS followed by distilled water, dried in a dust-free environment, and sputter coated with gold/palladium for visualization.

### Extraction of HA from cell culture supernatant and gel electrophoresis

The size of HA in the cell culture supernatant was determined by using gel electrophoresis. Cells (5 × 10^6^) were cultured in 50 ml cell culture medium, and the supernatant was collected 24 h after IL-10 treatment. The experimental procedure was similar to that of experiments performed for the analysis of PCM. DNase I treatment (2 h) and overnight Pronase E treatments were performed on supernatant to remove protein and DNA, which was followed by repeated phenol, phenol/chloroform (2×), and chloroform (2×) extractions (Sigma-Aldrich). Extract was then passed through Amicon spin concentration columns with a 10k molecular weight cutoff. This process yielded ∼50 µl of retentate that contained HA from 50 ml of medium. Gel electrophoresis was performed to determine the size of HA in the retentate. Agarose gel (0.5%) was poured and 20 µl of the retentate was resolved. DNA ladder and a commercially obtained HMW-HA of known molecular weight of 1.5 × 10^6^ kDa (EMD Millipore, Billerica, MA, USA) were used as controls. Gel was poststained with Stains-All (Sigma-Aldrich), and destaining was performed in 30% EtOH overnight to remove excess staining.

### Western blotting

Cells were homogenized, and 20 µg of protein was resolved by SDS-PAGE on a 4–20% Tris-glycine gel and transferred to nitrocellulose membrane by using the Iblot system (Thermo Fisher Scientific). Membranes were incubated with primary Abs against STAT3 [1:2000, (79D7) Rabbit mAb] or p-STAT3 [1:500, (Tyr705) (D3A7) Rabbit mAb] overnight at 4°C (Cell Signaling Technologies, Danvers, MA, USA), or β-actin (1:5000) for 30 min at room temperature (Abcam, Cambridge, MA, USA) and a horseradish peroxidase secondary Ab for 1 h at room temperature. Protein bands were visualized with ECL (Pierce, Rockford, IL, USA), followed by exposure to hyperfilm. Band densitometry was performed by using TotalLab software (New Castle, United Kingdom) and normalized against β-actin for loading controls.

### Immunocytochemistry

Cells were fixed in 1% paraformaldehyde for 20 min at room temperature (43) and incubated with α-STAT3 Ab [1:100 (79D7) rabbit mAb; Cell Signaling Technology’ overnight at 4°C, followed by Alexa Fluor goat anti-rabbit IgG secondary (Thermo Fisher Scientific). Cell nuclei were stained with POPO-3 Iodide (534/570) (1:400; Thermo Fisher Scientific). Cell filamentous actin was stained with Alexa Fluor phallodin (1:40; Thermo Fisher Scientific). Slides were mounted with Prolong Gold (Thermo Fisher Scientific) and imaged with confocal microscope (Carl Zeiss Zen 2009).

### Real-time quantitative PCR

Cells were lysed in 350 µl Buffer RLT (Qiagen, Valencia, CA, USA). Total RNA was isolated by using the RNeasy Micro Kit (Qiagen). cDNA was synthesized from 1 µg of RNA by using the High-Capacity cDNA Reverse Transcription Kit (Applied Biosystems, Foster City, CA, USA) following manufacturer protocols. SYBR green assays were designed to span intron/exon boundaries. Oligonucleotides were aligned against the mouse genome by Primer-BLAST (*http://www.NCBI.org*) to ensure specificity. Gene expression was assayed in triplicate by using 1/40 of the cDNA template and 300 nM of forward and reverse primers in a 25-µl Power SYBR Green PCR Master Mix reaction in the StepOne-Plus Real-Time PCR System (Applied Biosystems). Gene expression was normalized to mouse RPS20 gene expression. Relative expression values were calculated by using the ΔΔ*C_t_* method (44). Oligonucleotide primer sequences are listed in **Table 1**.

**TABLE 1.** Primer sequences for quantitative PCR validation

Oligonucleotide	Sequence, 5′–3′	Amplicon size (bp)	Intron spanning
Mo Has1 2F	CATGGGCTATGCTACCAAGT	77	3251
Mo Has1 2R	TCAACCAACGAAGGAAGGA		
Mo Has2 1F	AGTCATGTACACAGCCTTCA	85	11,587
Mo Has2 1R	GGCAGGGTCAAGCATAGTA		
Mo Has3 1F	CAGTGGACTACATCCAGAGGTG	73	2345
Mo Has3 1R	ACTCGAAGCATCTCAATGGT		
Mo Hyal1 1F	GAAAGTTTGGAGAATGAAGC	98	474
Mo Hyal1 1R	TCAGGAAGAGAGTAGAGATG		
Mo Hyal2 1F	TCTTCACGCGTCCCACATA	79	421
Mo Hyal2 1R	GCACTCTCACCGATGGTAGA		
Mo KIAA1199 1F	CTCAGCTGAAGACAAAAGAC	77	1F split by 187-bp intron
Mo KIAA1199 1R	ATTCCGAAGGTGGAAGAAG		
Mo Stat3 1F	AAGTCAGGTTGCTGGTCAAA	85	Yes
Mo Stat3 1R	GCAACATCCCCAGAGTCTTT		
Mo Rps20 F	GCTGGAGAAGGTTTGTGCG	100	182 and 252
Mo Rps20 R	AGTGATTCTCAAAGTCTTGGTAGGC		

F, forward; R, reverse.

### ELISA

Cell culture medium was collected after treatments, and HA levels were determined by using the HA Test Kit (Corgenix, Broomfield, CO, USA). IL-10 levels in skin and wounds were measured in tissue homogenate by using the Quantikine IL-10 ELISA Kit (R&D Systems, Minneapolis, MN, USA). Data were normalized against total protein in each sample and calculated by using the Coomassie Plus protein assay (Thermo Fisher Scientific).

### IL-10 receptor 1 neutralization

IL-10 receptor 1 (IL-10R1; 2 µg/ml) Ab [rat monoclonal (1B1.3a)] or IgG control was added (Abcam) to the conditioned culture medium for 20 min before addition of treatments.

### Lentiviral short hairpin RNA-knockdown cells

Stable cell lines of *STAT3^−/−^* AFB were generated by using lentivirus-based short hairpin RNA (shRNA). Gene sequence–specific shRNA clones were constructed within the lentivirus plasmid vector pLKO.1-puromyocin, which was obtained *via* the Lenti-shRNA Library Core facility (Cincinnati Children's Hospital Medical Center, Cincinnati, OH, USA) from Sigma-Aldrich. Five clones were obtained (**Table 2**). Primary AFBs (passage 5) were transduced with individual clones and a combination of all 5 clones at a multiplicity of infection of 1:10 for 24 h in serum-free medium that contained polybrene (8 µg/ml). Cells were washed and allowed to recover in normal DMEM with 10% BGS and then selection was achieved by inclusion of 1 ug/ml puromycin in culture medium. Transduction negative control was a Lenti-shRNA scramble construct. In all experiments, control and knockdown cells were matched to receive a similar number of lentiviral particles to control for viral effects. Modified gene expression and protein expression were verified by quantitative PCR and Western blot for STAT3, respectively, as explained above.

**TABLE 2.** DNA sequences of STAT3 in lenti-shRNA constructs

Protein	Construct	DNA sequence
STAT3	2-1	CCGGCCTAACTTTGTGGTTCCAGATCTCGAGATCTGGAACCACAAAGTTAGGTTTTTG
	2-2	CCGGCGACTTTGATTTCAACTACAACTCGAGTTGTAGTTGAAATCAAAGTCGTTTTTG
	2-3	CCGGCCTGAGTTGAATTATCAGCTTCTCGAGAAGCTGATAATTCAACTCAGGTTTTTG
	2-4	CCGGCACCATTCATTGATGCAGTTTCTCGAGAAACTGCATCAATGAATGGTGTTTTTG
	2-5	CCGGGCAGGTATCTTGAGAAGCCAACTCGAGTTGGCTTCTCAAGATACCTGCTTTTTG

### Animal studies

To validate *in vitro* results, we conducted a series of *in vivo* experiments by using an excisional cutaneous wound model in mice that was controlled for contracture to more closely represent wound repair by secondary intension. All animal procedures were approved by the Cincinnati Children’s Hospital Medical Center Institutional Animal Care and Use Committee.

### Lentiviral transfer to overexpress IL-10 in murine wounds

Each animal received 2 wounds in the same anatomic locations on bilateral flanks. One wound was treated with IL-10, GPF, or PBS, and the other paired wound was always untreated and served as an internal control for that animal. To investigate the *in vivo* effects of IL-10, 1 × 10^6^ transducing units (TU) of lentivirus that expressed IL-10 (lenti–IL-10) or GFP (lenti-GFP) (31) or a PBS control were injected intradermally in the dorsal skin of 8- to 10-wk-old mice at the anticipated wound sites in the bilateral flanks and labeled with India ink. To allow for transgene expression, skin wounds were created 4 d after injection.

### Wounding and wound care

Mice were anesthetized with isoflurane inhalation (0.5 ml titrated). Dorsal skin was shaved and prepared by scrubbing alternately with isopropyl alcohol and betadine. Full thickness excisional murine wounds were created at injection sites by using a 4-mm punch biopsy (Miltex, Plainsboro, NJ, USA) and were stented. Wounds were covered with a sterile adhesive dressing (Tegaderm; 3M, St. Paul, MN, USA) and were allowed to heal.

### Inducible skin-specific ubiquitin C STAT3^−/−^ transgenic murine model

Cre-expressing mice that were driven by the human ubiquitin C (UBC) promoter [B6.Cg-Tg (UBC-Cre/ERT2) 1Ejb/ J; The Jackson Laboratory] were bred with mice that contained loxP-flanked STAT3^flox/flox^ sequence [on FVBN background; gifted by Dr. Jeffrey Whitsett (Cincinnati Children’s Hospital, Cincinnati, OH, USA)]. STAT3^flox/flox^ mice used in this study were backcrossed to C57BL/6J for at least 8 generations. Double transgenic phenotype was confirmed by genotyping the mice. DNA was generated from the tail or ear of experimental mice. PCR amplification was performed for Cre-ERT2 using the primers Cre8000 (5′–TTGAAGCAACTCATCGATTGATTT–3′) and ERT28640 (5′–AGAGCAAGTTAGGAGCAAACAG–3′), and STAT3^flox/flox^ genes using the primers Stat3-1 (5′–CCTGAAGACCAAGTTCATCTGTGTGA–3′) and Stat3-2 (5′–CACACAAAGCCATCAAACTCTGGTCTC–3′). Amplification was performed by using Platinum PCR SuperMix High Fidelity (Thermo Fisher Scientific) with 300 nM of primers in the following thermal cycling protocol: 5 min initial denaturation at 95°C, 35 cycles of 30 s at 93°C, 1 min at 65°C, and 2 min at 72°C, followed by a final extension of 7 min at 72°C. 4-Hydroxy tamoxifen (4-OHT; 10 mg/ml in sterile vegetable oil, 150 µl per mouse) was topically applied on the shaved dorsal skin every day for 7 d to activate Cre-mediated recombination and STAT3 deletion. Vegetable oil only was included as a vehicle control group. To determine the extent of STAT3 knockdown in the transgenic mice after 4-OHT administration for 7 d, dorsal skin snips from the treatment area were collected and homogenized in ice-cold Tris-buffered saline that contained 1% igepal, 0.1% protease inhibitor cocktail, 20 mM NaF, 1 mM Na3VO4, and 0.1% Tween-20 (all from Sigma-Aldrich), and Western blot and band densitometry analysis were performed to check protein levels of STAT3. STAT3 knockdown of the treated skin was also confirmed by immunohistochemical analysis by using Abs against STAT3 as previously described. After STAT3 knockdown was confirmed, dorsal skin was pretreated with lenti–IL-10, lenti-GFP, or PBS, and excisional wounding was performed similar to the control group as previously described.

### Fibroblast-Col1a2 STAT3^−/−^ and keratinocyte-K14 STAT3^−/−^ murine models

STAT3^flox/flox^ mice were similarly bred to either tamoxifen-inducible Col1a2 Cre-ERT mice [B6.Cg-Tg(Col1a2-Cre/ERT)7Cpd/J; The Jackson Laboratory] or Krt14 Cre-ERT mice [Tg(KRT14-Cre/ERT)20Efu/J; The Jackson Laboratory] to obtain fibroblast-specific or keratinocyte-specific inducible *STAT3^−/−^* mice, respectively.

### Syngeneic fibroblast transplant

Primary AFBs (passages between 5 and 10) were transduced with either lenti-GFP or lenti–IL-10 (1 × 10^6^ TU/ml) at a multiplicity of infection of 1:10 for 72 h. Transduced cells (7.5 × 10^5^) were reconstituted in 35 µl of PBS and transplanted into each 4-mm murine dorsal wound immediately after wounding. Live imaging of murine wounds with syngeneic fibroblast transplantation and wounds without any treatment was performed at d 1, 5, and 30 postwounding to determine green fluorescence using an IVIS Imaging System 100 (PerkinElmer, Waltham, MA, USA).

### HA-knockout murine model

HA knockdown was induced in mice by 4-MU. Six-week-old C57BL/6J mice were fed 10–20 mg 4-MU/g body weight/mouse/d pelleted into rodent chow for 2 wk, and levels of HA in the dorsal skin were analyzed to confirm HA knockdown. Excisional wounding was performed and mice were maintained on 4-MU diet throughout the wound healing period until wounds were harvested.

### Tissue analysis

Wounds were harvested from different animals. We included 4–6 wounds from different animals per treatment group per time point. Wounds were harvested at d 1, 3, and 5 postwounding. Half were processed for RNA isolation (mini kit; Qiagen), and HAS1–3 gene expression was analyzed by quantitative real-time PCR. The other half were homogenized in 250 µl of buffer (PBS with 0.1% protease inhibitor cocktail and no detergent), and HA synthesis was analyzed by ELISA (Corgenix). Wounds (*n* = 4–6/treatment group from different mice) were also harvested at d 28, fixed in 10% neutral buffered formalin, and embedded in paraffin to determine the effects of IL-10 on wound healing/scarring phenotype. Wound sections (5 µm) were cut. Repair tissue morphology and ECM architecture were determined by hematoxylin and eosin staining and Masson’s Trichrome staining (Polyscientific, Bay Shore, NY, USA). Scar area and dermal appendages per ×40 high-power field in the dermis of each of the wounds were measured. Images were obtained by using a Nikon Eclipse microscope, and image analysis was performed by using the Nikon Elements software (Nikon Instruments, Melville, NY, USA).

### Statistical analysis

Statistical analysis of data was performed by using ANOVA, followed by Bonferroni *post hoc* tests or Student’s *t* test when appropriate. Data are expressed as means ± sd. A value of *P* < 0.05 was considered statistically significant.

## RESULTS

### IL-10 recapitulates the FFB phenotype of HA-rich PCM in AFBs

**Figure 1.**
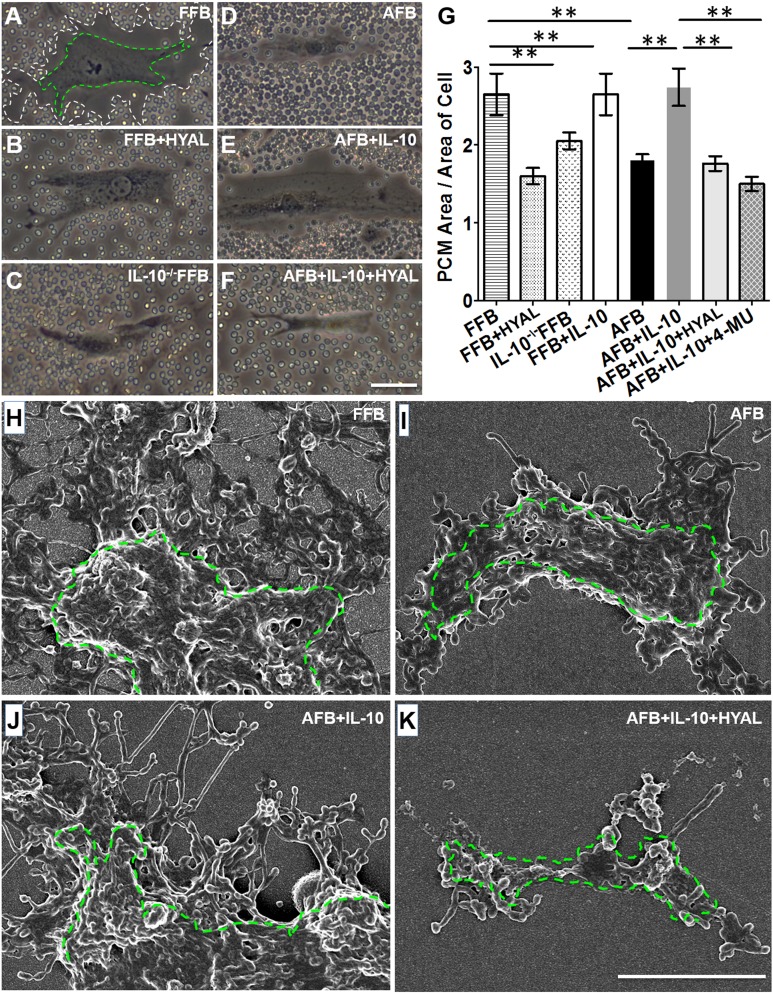
IL-10 induces a fetal-type HA-enriched PCM in AFBs. *A*–*F*) Living fibroblasts were imaged with phase contrast to visualize cell bodies, and the area occupied by the pericellular HA coat after 24 h of treatment was assessed by the exclusion of red blood cells from the cell perimeter. White and green dotted lines were artificially drawn in *A* to delineate the margin of the HA coat and cell body, respectively. Representative cells are shown for FFBs and AFBs under different treatment conditions: FFBs (*A*), FFBs treated with HYAL (*B*), IL-10^−/−^ FFBs (*C*), AFBs (*D*), AFBs treated with IL-10 (*E*), and AFBs treated with IL-10 and HYAL (*F*). *G*) We quantified the ratio of the means ± sd of 3 experiments (20 cells analyzed per experiment). *H*–*K*) Scanning electron microscopy was performed to demonstrate HA cable-like structures on the cell body outlined by green dotted line. Representative cells are shown for different cell types under different treatment conditions: FFBs (*H*), AFBs (*I*), AFBs treated with IL-10 (*J*), and AFBs treated with IL-10 and HYAL (*K*). See also Supplemental Fig. S1. Scale bar, 50 μm (*A*–*F*), 5 μm (*H*–*K*). **P < 0.01 by ANOVA and *post hoc* Bonferroni tests.

We first asked whether IL-10 induces an HA-enriched PCM by AFBs that would be similar to FFBs in area and morphology of its ultrastructure. To assess the size of the PCM, we performed a particle exclusion assay on FFBs (E14.5) and AFBs (8–10 wk) that were isolated from C57BL/6J mice. Representative images for each condition are shown in **Fig. 1*A*–*F*** and quantified in Fig. 1*G*. FFBs produced a significantly larger PCM compared with that of AFBs (Fig. 1*A*, *D*; *P* < 0.001). HYAL treatment of the FFBs significantly reduced the PCM (*P* < 0.01), which indicated that the PCM associated with FFBs was primarily composed of HA (Fig. 1*B*). We then examined the PCM formation in FFBs that were isolated from *IL-10^−/−^* transgenic mice, which also demonstrated a significantly reduced PCM compared with FFBs from wild-type control mice at the same gestational age (Fig. 1*C*; *P* < 0.01). This result suggested that IL-10 is important to the formation of HA-rich PCM in FFBs. Next, to determine whether IL-10 could recapitulate the PCM phenotype of FFBs in AFBs, AFBs were treated with 200 ng/ml IL-10 for 24 h. IL-10 supplementation significantly increased the size of the PCM compared with control AFBs (*P* < 0.01) to levels that were not statistically different than in FFBs (Fig. 1*E*). Furthermore, HYAL treatment significantly attenuated the IL-10–induced PCM formation in AFBs, which suggested that the PCM induced by IL-10 in AFBs was mainly composed of HA (Fig. 1*F*). Of note, when HA is digested by treatment with HYAL, other HA binding proteins that stick to HA may also be removed from the PCM. Therefore, we measured the effect of IL-10 on HA-rich PCM by using 4-MU treatment, which showed a similar attenuation of the effect of IL-10 on PCM formation by AFBs. Furthermore, our data demonstrated that IL-10 induced a fetal-type HA-enriched PCM in AFBs, regardless of the quantity differences of primary receptor IL-10R1 between FFBs and AFBs (Supplemental Fig. S1*A*, *B*). There were qualitative differences in HA produced by FFBs, compared with HA produced by AFBs, as determined by preliminary sizing of HA that was extracted from cell culture supernatant using gel electrophoresis (Supplemental Fig. S1*C*).

We next used scanning electron microscopy to assess ultrastructural differences between the PCM that was formed by FFBs, and that induced by IL-10 in AFBs. PCM produced by FFBs demonstrated rope-like structures that extended from the cell wall (Fig. 1*H*) that were not present in PCM produced by AFBs (Fig. 1*I*). IL-10 induced similar rope-like structures in AFBs (Fig. 1*J*), which were abrogated by addition of HYAL (Fig. 1*K*), which again suggested that these structures were composed of HA.

### IL-10 regulates HA *via* STAT3 and the differential regulation of HASs and HYALs

**Figure 2.**
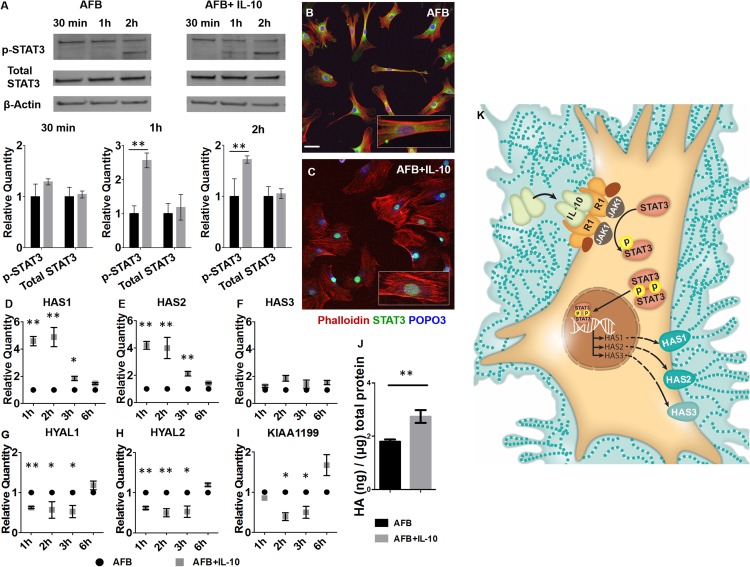
IL-10 induction of HA synthesis *via* phosphorylation and nuclear translocation of STAT3, and the differential regulation of HASs and HYALs. *A*) Western blots of p-STAT3 and total STAT3 in AFBs in response to IL-10 stimulation at 30 min and 1 and 2h. β-Actin was analyzed as a loading control. Densitometric analysis of means ± sd of 3 experiments is shown in the bar plots. *B*, *C*) Cells were labeled with a STAT3 Ab and the representative images, with zoomed in insets, for the perinuclear STAT3 staining in control AFB (*B*) as well as the nuclear translocation of activated STAT3 after AFB stimulation with IL-10 for 60 min (*C*) are shown. *D*–*I*) Gene expression of HAS1 (*D*), HAS2 (*E*), HAS3 (*F*), HYAL1 (*G*), HAS2 (*H*), and KIAA1199 (*I*) in IL-10–stimulated AFBs at 1, 2, 3, and 6 h after stimulation with IL-10. Bar plots represent mean ± sd for 2 experiments. *J*) HA ELISA for AFBs 24 h after IL-10 (200 ng/ml) stimulation. Means ± sd of 3 experiments. *K*) Schematic illustration of the IL-10 pathway (see also Supplemental Fig. S2). Scale bar, 50 μm. *P < 0.05, **P < 0.01 by ANOVA and *post hoc* Bonferroni tests.

We elucidated the signaling pathway that is involved in IL-10–mediated HA production by AFBs. Given the established role for STAT3 in IL-10R signaling, we investigated the effect of IL-10 stimulation on pSTAT3 at various time points in AFBs. IL-10 did not affect total levels of STAT3 protein, which remained unchanged at all timepoints after treatment. In contrast, IL-10 supplementation significantly increased p-STAT3 at 1 and 2 h compared with controls (*P* < 0.01; **Fig. 2*A***; refer to Supplemental Fig. S2 for complete Western blots). Next, to determine whether IL-10 induced nuclear translocation of p-STAT3, we used STAT3 immunohistochemistry and confocal microscopy. IL-10 treatment resulted in increased nuclear localization of STAT3 at 1 h compared with the untreated control AFBs, in which STAT3 remained perinuclear in the cytoplasm of the cell (Fig. 2*B*, *C*).

We then investigated the effect of IL-10 on HA synthesis and degradation by AFBs by using quantitative real-time PCR for transcriptional analysis of the 3 HASs and the known HYALs in skin. IL-10 significantly increased HAS1 and HAS2 gene transcription at 1, 2, and 3 h (*P* < 0.05) compared with untreated AFB (Fig. 2*D*, *E*). In contrast, IL-10 did not have an influence on HAS3 transcription at any of the assessed time points (Fig. 2*F*). IL-10 stimulation reduced the transcription of HYAL1, HYAL2, and KIAA1199 at 1, 2, and 3 h compared with control AFBs (Fig. 2*G*–*I*). The net effect of the differential regulation of HASs and HYALs by IL-10 resulted in increased formation of PCM (as shown in Fig. 1) and increased levels of HA compared with untreated AFBs (*P* < 0.01; Fig. 2*J*; pathway is summarized schematically in Fig. 2*K*).

### IL-10R1 neutralization abrogates the effect of IL-10 on STAT3 nuclear translocation, HAS gene expression, and formation of HA

**Figure 3.**
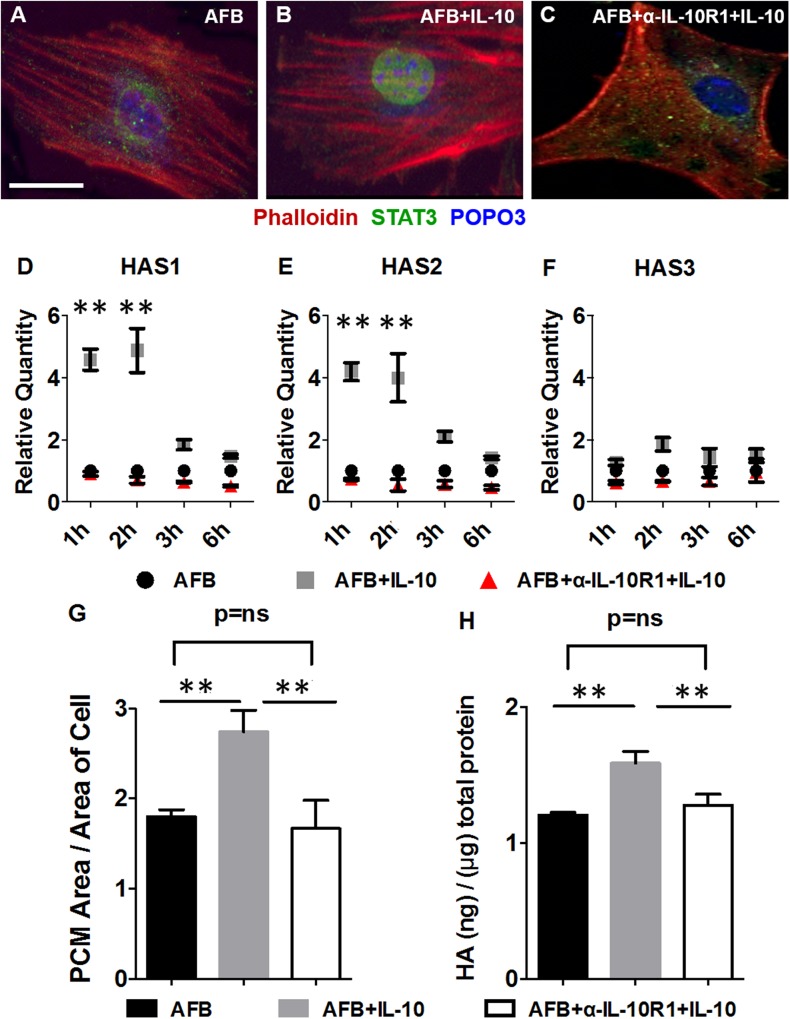
Competitive inhibition of IL-10 binding to IL-10R1 abrogates the effect of IL-10 on STAT3 nuclear translocation and HA-rich PCM formation. *A*–*C*) Immunofluorescence staining for STAT3 translocation in AFBs in response to IL-10 with (*C*) or without (*B*) addition of an α-IL-10R1 Ab that inhibits IL-10 binding, along with control (*A*). Data are for 60 min after stimulation. *D*–*F*) Expression of HAS1 (*D*), HAS2 (*E*), and HAS3 (*F*) mRNA in response to IL-10 treatment at 1, 2, 3, and 6 h in the setting of α-IL-10R1 addition. Bar plots represent means ± sd for 2 experiments. *G*) Particle exclusion assay assessment of PCM area of AFBs at 24 h after IL-10 (200 ng/ml) treatment in the setting of α-IL-10R1 addition. *H*) HA ELISA of supernatants from AFBs stimulated with IL-10 with or without α-IL-10R1 treatment. Scale bar, 50 μm. ns, not significant. **P < 0.01 by ANOVA and *post hoc* Bonferroni tests.

To investigate whether the effects of IL-10 on HA were mediated *via* its primary receptor IL-10R1, we used an IL-10R1 Ab that competitively inhibits IL-10 binding (41). IL-10R1 neutralization indeed abrogated the effects of IL-10 on STAT3 nuclear translocation in AFBs (**Fig. 3*A*–*C***). By using quantitative real-time PCR, we demonstrated that the effects of IL-10 on HAS1 and HAS2 gene expression in AFBs were also abrogated in the presence of an IL-10R1 Ab (Fig. 3*D*–*F*). Furthermore, we demonstrated that IL-10R1 neutralization with IL-10R1 Ab abrogated the effect of IL-10 on PCM and HA production to levels that were statistically similar to untreated control AFBs (*P* < 0.01; Fig. 3*G*, *H*). Addition of IgG control had no effect. These results suggested that the primary receptor IL-10R1 was critical for mediating the effects of IL-10 on HA synthesis.

### IL-10 regulates HAS gene expression and HA-rich PCM formation *via* STAT3

**Figure 4.**
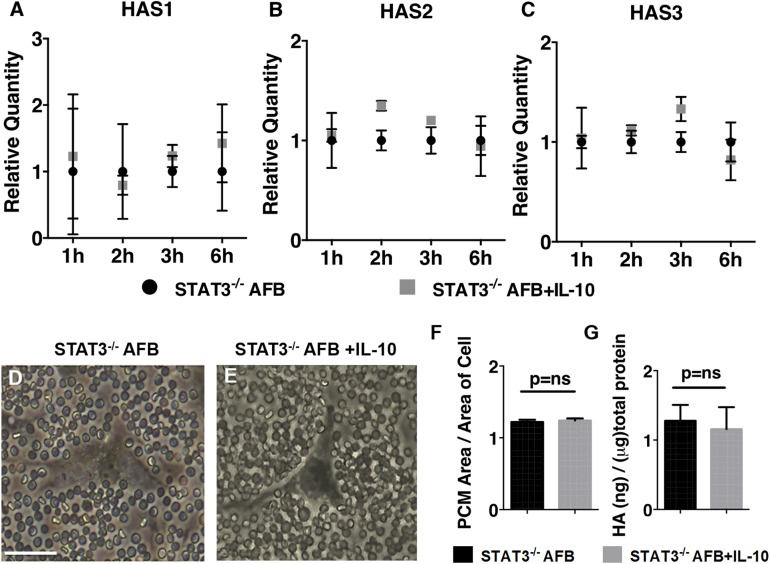
IL-10–induced effects on regulation of HAS1-3 and HA-rich PCM are mediated *via* STAT3 signaling. *A*–*C*) Time course for mRNA expression of HAS1 (*A*), HAS2 (*B*), and HAS3 (*C*) in STAT3^−/−^ AFBs with or without IL-10 treatment. Bar plots represent means ± sd of 2 experiments. *D*, *E*) Pericellular HA coat assessment *via* particle exclusion assay in STAT3^−/−^ AFBs (*D*) and STAT3^−/−^ AFBs treated with IL-10 (*E*). *F*) PCM ratio and means ± sd of 3 experiments (20 cells analyzed per experiment) are shown for STAT3^−/−^ AFBs and STAT3^−/−^ AFBs treated with IL-10 (200 ng/ml) for 24 h. *G*) HA levels in culture medium of STAT3^−/−^ AFBs and STAT3^−/−^ AFBs treated with IL-10 (200 ng/ml) for 24 h. Means ± sd of 3 experiments (see also Supplemental Fig. S3). Scale bar, 50 μm. ns, not significant.

We asked whether induction of HA-rich PCM formation by IL-10 in AFBs is STAT3 dependent. To study this, we generated stable *STAT3^−/−^* AFB cell lines by using shRNA gene silencing. STAT3 was significantly knocked down in AFBs with clone 1 used in this study, as demonstrated by reduction of expression at both the gene level (approximately 75% reduction in STAT3 expression as quantified by quantitative RT-PCR; *P* < 0.001) and at the protein level (approximately 90% reduction in STAT3 expression as quantified by Western blot analysis; *P* < 0.001; Supplemental Fig. S3). In *STAT3^−/−^* AFBs, IL-10 stimulation did not alter HAS1-2 gene expression, which was observed in control AFBs, suggesting that this effect of IL-10 was mediated *via* STAT3 (**Fig. 4*A*–*C***). Next, to determine the role of STAT3 in IL-10–induced formation of HA-rich PCM in AFBs, *STAT3^−/−^* AFBs were treated with IL-10, and PCM was measured. IL-10 treatment did not increase PCM formation (Fig. 4*D*–*F*) or HA production (Fig. 4*G*) in *STAT3^−/−^* AFBs, which suggested that this effect of IL-10 in control AFB was indeed mediated *via* STAT3.

### Effect of IL-10 on regenerative wound healing *in vivo* is dependent on HA

To evaluate the *in vivo* relevance of our findings and to further test our hypothesis that IL-10 promotes the formation of an HA-rich ECM, which results in regenerative wound healing in postnatal wounds, we performed an *in vivo* wound-healing experiment. Dorsal wounds were induced in 8- to 10-wk-old C57BL/6J wild-type control mice with or without overexpression of IL-10 or control treatments. Previous observations that used either adenoviral (32) or lentiviral (31) vectors, which have short-term *vs*. long-term transgene expression, respectively, or recombinant protein (38), suggest that a brief, early, sustained exposure of the wound to increased levels of IL-10 is required for recapitulating scarless phenotype in postnatal wounds. We used lentiviral vectors in this study to achieve IL-10 overexpression. It has been previously demonstrated that efficient transduction—with transgenic protein expression detected at the base of wounds within 72 h after injection, and sustained expression for at least 7 d postwounding—can be achieved with lentiviral vectors (31). This also enabled us to overcome the limitations of recombinant IL-10 therapy, which requires repetitive dosing as a result of the shorter half-life of the recombinant proteins or the enhanced inflammation associated with adenoviral vectors.

**Figure 5.**
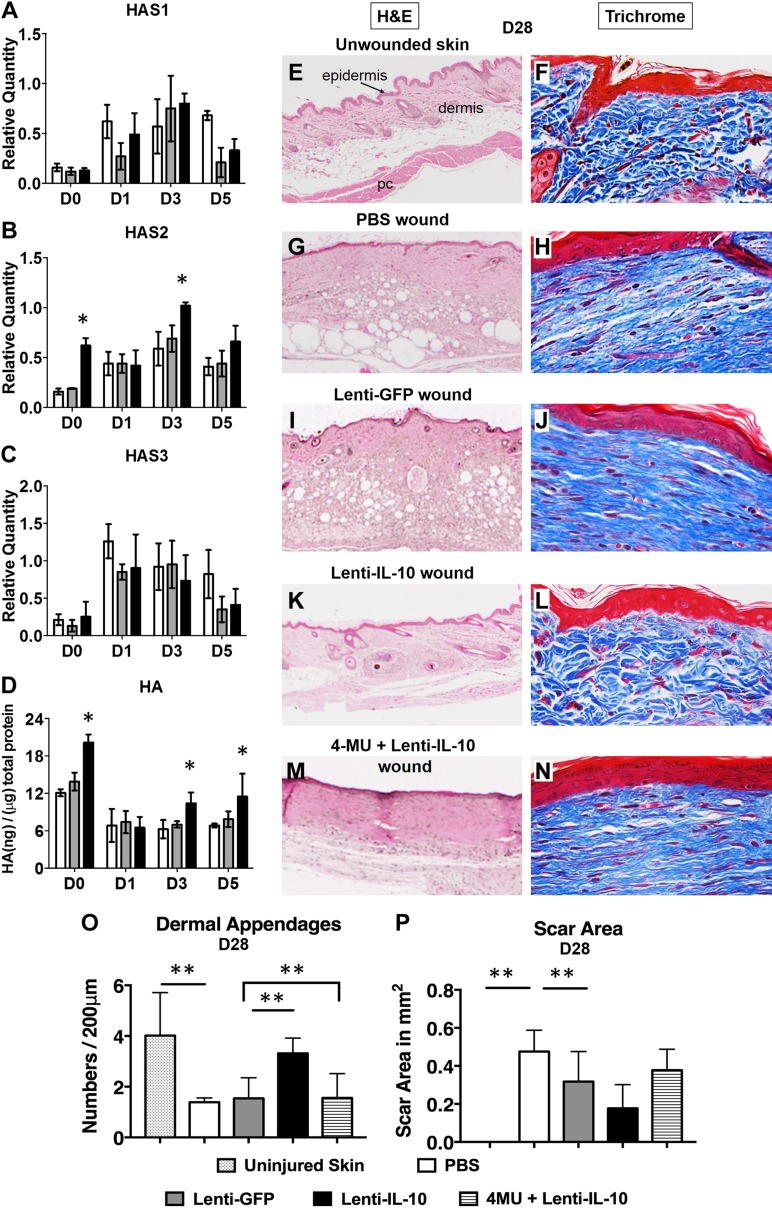
HAS2 and HA are up-regulated in IL-10–treated *in vivo* wounds at 3 d postwounding, and the effect of IL-10 on regenerative wound healing in postnatal wounds is dependent on HA. *A*–*D*) mRNA expression of HAS1 (*A*), HAS2 (*B*), and HAS3 (*C*), and HA levels (*D*) in homogenized pretreated skin at d 0 and wound tissue at d 1, 3, and 5 postwounding. *E*–*L*) Representative histology for uninjured murine skin and murine wounds treated with lentiviral IL-10 and controls at 28 d postwounding. Images are for hematoxylin and eosin (H&E) staining (*E*, *G*, *I*, *K*; images captured with a ×4 objective; scale bar, 500 μm) and Masson’s trichrome staining (*F*, *H*, *J*, *L*; images captured with a ×40 objective; scale bar, 100 μm) of the repair tissue. *M*, *N*) Representative H&E staining (*M*) and trichrome staining (*N*) of a wound treated with lentiviral IL-10 in conjunction with 4-MU, an HAS inhibitor, at d 28 postwounding. *O*, *P*) Bar graphs show 2 elements for how we quantified scar formation and wound regeneration, including the dermal appendages (*O*) and scar area (*P*), respectively (*n* = 4–6 animals per group). pc, panniculus carnosus muscle layer. *P < 0.05, **P < 0.01 by ANOVA and *post hoc* Bonferroni tests.

To determine the role of IL-10 in the regulation of HAS1-3 and HA synthesis under normal wound-healing conditions in C57BL/6J wild-type mice, normal skin with 4 d of pretreatment with lenti–IL-10, lenti-GFP, or PBS (d 0) and cutaneous wounds (postwounding d 1, 3, and 5) were harvested at various timepoints during the initial phase after wounding and were examined. Lenti–IL-10 administration resulted in a significant increase in the expression of IL-10 protein in the pretreated area (lenti–IL-10: 135.25 ± 17.65 pg of IL-10/mg total protein *vs*. undetectable IL-10 levels in lenti-GFP and PBS groups). Day 0 data demonstrated that pretreatment of skin with lenti–IL-10 overexpression does not affect HAS1 or HAS3 gene expression compared with pretreatment with PBS or lenti-GFP, but it significantly increased baseline HAS2 gene expression and HA levels in the skin. Lenti–IL-10 overexpression significantly up-regulated HAS2 gene expression by quantitative PCR at d 3 postwounding (*P* < 0.05; **Fig. 5*B***), which corresponded to a significant increase in HA synthesis by ELISA at d 3 and 5 postwounding (*P* < 0.05; Fig. 5*D*) compared with lenti-GFP and PBS control treatments. HAS1 (Fig. 5*A*) and HAS3 (Fig. 5*C*) gene expression were not significantly different compared with control treatments.

Next, to validate the *in vivo* ability of IL-10 to induce regenerative wound healing, we examined normal skin and wounds with various treatments, including PBS, lenti–IL-10, and lenti-GFP at 28 d postwounding. Representative images for hematoxylin and eosin and Masson’s trichrome staining are shown in Fig. 5*E*–*L*. Unwounded native skin has a characteristic epidermal topography with distinct cell layers and a dermis that is comprised of appendages, including hair follicles and sweat glands, and the underlying panniculus carnosus muscle layer (Fig. 5*E*). ECM in the dermis of uninjured skin also has a unique pattern of collagen fibers that are packed in a basket-weave form, as shown in blue in the Masson’s trichrome staining (Fig. 5*F*). PBS-treated (Fig. 5*G*, *H*) and lenti-GFP-treated (Fig. 5*I*, *J*) wounds demonstrated a characteristic scar formation with flattened epidermis and a dermis that was remodeled with thick parallel bundles of collagen in the ECM, with a lack of formation of dermal appendages. In contrast, lenti–IL-10–treated wounds (Fig. 5*K*, *L*) demonstrated a restoration of dermal appendages and collagen pattern in the repair wound that was nearly indistinguishable from the surrounding normal uninjured skin.

To further determine whether this effect of IL-10 was dependent on HA, we used an HAS inhibitor, 4-MU, that has been shown to suppress HA production by all 3 HASs (45). C57BL/6J mice were fed 4-MU chow or control chow. Dorsal wounds were created similarly and were examined at 28 d postwounding (46). 4-MU administration did not affect normal scar formation in control mice. In lenti–IL-10–treated wounds without the ability to synthesize HA as a result of 4-MU, the regenerative effects of IL-10 were abrogated, and the wound repair defaulted back to the characteristic scar formation with disorganized collagen bundles, lack of dermal appendages, and thickened epidermis (Fig. 5*M*, *N*), similar to scar formation in lenti-GFP and PBS wounds.

Quantification of dermal appendages and scar area in the different treatments are shown in Fig. 5*O*, *P*, respectively. Together, these data demonstrate that IL-10’s ability to promote regenerative wound healing *in vivo* is HA dependent.

### IL-10 effects in regenerative wound healing are primarily mediated by a STAT3-dependent mechanism in fibroblasts

**Figure 6.**
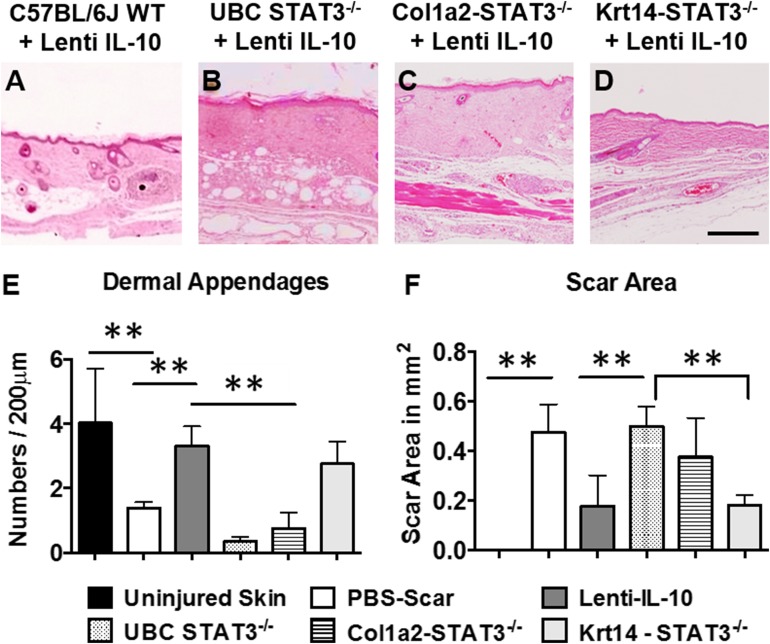
IL-10 effects in regenerative wound healing are fibroblast dependent. *A*–*D*) Hematoxylin and eosin staining of wounds in different control (*A*), skin-specific (*B*), fibroblast-specific (*C*), and keratinocyte-specific (*D*) STAT3^−/−^ mice treated with lentiviral IL-10, all at 28 d. Images are captured with a ×4 objective. *E*, *F*) Bar graphs show 2 elements for how scar formation and wound regeneration was quantified, including the dermal appendages (*E*) and scar area (*F*), respectively (*n* = 4–6 animals per group). See also Supplemental Fig. S4. Scale bar, 250 μm. **P < 0.05, by ANOVA and *post hoc* Bonferroni tests.

To determine *in vivo* whether the effect of IL-10 on regenerative wound healing was dependent on STAT3, we created tamoxifen-inducible skin-specific (UBC Cre) *STAT3^−/−^* mice (Supplemental Fig. S4). Next, to determine whether dermal fibroblasts are the key cellular mediator of the effects of IL-10 on regenerative wound healing, we created tamoxifen-inducible fibroblast (Col1a2-Cre) and keratinocyte (K14-Cre)-specific *STAT3^−/−^* mice (Supplemental Fig. S4) and compared wound healing in these animals with that observed in skin-specific *STAT3^−/−^* mice. Dorsal wounds were similarly investigated at d 28 postwounding to determine scar formation in all 3 models. All animals survived the procedures well, similar to C57BL/6J wild-type controls, and demonstrated that neither 4-OHT treatment nor skin-, fibroblast-, or keratinocyte-specific STAT3 knockdown affect the baseline ability of these mice to heal cutaneous wounds. Control treatments, including lenti-GFP overexpression and PBS treatment, in all 3 knockdown models healed with a characteristic scar. In control murine wounds, overexpression of lenti–IL-10 resulted in regenerative wound healing, with a significant reduction in scar area and an increase in dermal appendages (**Fig. 6*A***). In both the skin- (Fig. 6*B*) and fibroblast-specific (Fig. 6*C*) STAT3-knockout mice, the effects of IL-10 were abrogated, with the scar area and dermal appendages at levels consistent with normal scar formation in PBS-treated wounds (Fig. 6*E*, *F*). Of interest, when signaling *via* STAT3 was only knocked down in the keratinocytes, the effect of IL-10 on wound healing was maintained (Fig. 6*D*), with improvements in scar area and dermal appendages statistically similar to lenti–IL-10–treated wounds in control animals. These results indicate that the effects of IL-10 on regenerative wound healing in postnatal wounds are primarily mediated by fibroblasts.

### Transplant of syngeneic dermal fibroblasts that overexpress IL-10 recapitulate the regenerative effects of IL-10 in STAT3^−/−^ wounds *in vivo*

**Figure 7.**
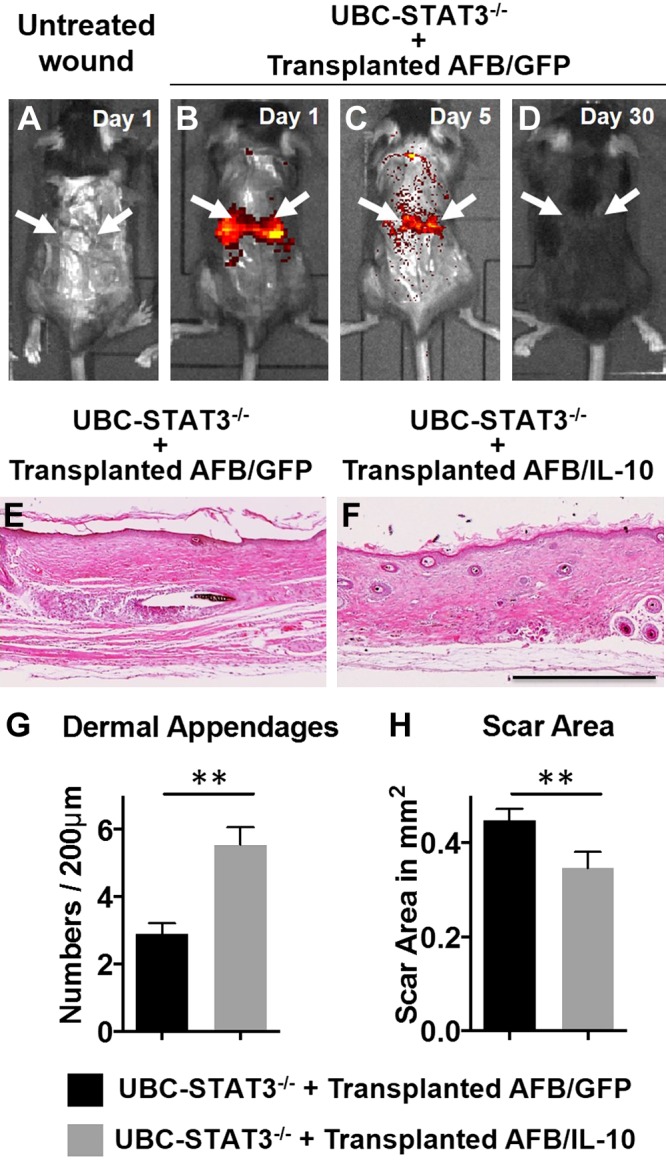
Syngeneic dermal fibroblast transplantations recapitulate the regenerative effects of IL-10 in STAT3^−/−^ wounds *in vivo.* *A*–*D*) Representative image from IVIS *in vivo* imaging of murine untreated wounds at d 1 postwounding (*A*) and wounds treated with syngeneic genetically modified adult fibroblasts at d 1 (*B*), d 5 (*C*), and d 30 (*D*) postwounding. *E*, *F*) Hematoxylin and eosin staining of d 28 wounds in skin-specific STAT3^−/−^ mice treated with syngeneic genetically modified AFBs, which express either GFP (*E*) or IL-10 (*F*). Images are captured with a ×4 objective. *G*, *H*) Dermal appendages (*G*) and scar area (*H*) for skin-specific STAT3^−/−^ wounds with different AFBs transplant treatments. Arrows indicate the wounds (*n* = 3 animals per group). Scale bar, 500 μm. **P < 0.01 by Student’s *t* test.

To confirm the role of fibroblasts as critical mediators of the effects of IL-10 described here, we performed a gain-of-function experiment. For this, we used a syngeneic fibroblast transplant model in wounds in skin-specific *STAT3****^−/−^*** mice. Fibroblasts were transfected *in vitro* with lenti–IL-10 or lenti-GFP, then transplanted topically into wounds in syngeneic skin-specific *STAT3^−/−^* recipient mice. Therefore, the only cells that were capable of responding to IL-10 in the wound were the transplanted fibroblasts. *In vivo* imaging of murine wounds under excitation at 465 nm wavelength demonstrated fluorescence detection that was specific to the wounds that received fibroblast transplantation at d 1 compared with no fluorescence detection in the untreated wounds (**Fig. 7*A*, *B***). By d 5 postwounding, detection intensity in fibroblast-transplanted wounds reduced (Fig. 7*C*), and no fluorescence was detected in transplanted wounds at d 30 postwounding (Fig. 7*D*). Skin-specific *STAT^−/−^* murine wounds transplanted with fibroblasts that expressed GFP healed with characteristic scar formation, with an increased scar area and reduced appendages (Fig. 7*E*). In comparison, wounds that were transplanted with fibroblasts that expressed IL-10 (Fig. 7*F*) demonstrated a significant reduction in scar area and a significant increase in dermal appendages (Fig. 7*G*, *H*). However, the effect on scar reduction was not as optimal as with lenti–IL-10 overexpression. These data support the primary role of fibroblasts as the key mediator of the effects of IL-10 on regenerative wound healing.

## DISCUSSION

We report here that treating AFBs with IL-10 recapitulates a fetal-like phenotype in these cells. AFBs treated with IL-10 produce pericellular HA and mediate regenerative healing in postnatal wounds in a STAT3-dependent manner (this pathway is summarized schematically in Fig. 2*K*). We propose that the capacity of IL-10 to shape ECM in wounds complements the well-established immunologic effects of this cytokine in ways that together promote regenerative healing, as observed in fetal tissues.

Our data indicate that IL-10 has direct effects on AFBs. By using both *in vitro* cell culture models and *in vivo* wound models, we found that IL-10 promotes production of HA mediated by STAT3 in these cells. This conclusion is supported by data that were generated with several novel cell lines and mouse strains first reported here. These include tamoxifen-inducible, Cre-mediated skin-, keratinocyte-, and fibroblast-specific models for STAT3 knockdown to selectively inhibit IL-10 signaling. Further support for this model comes from experiments with syngeneic AFB transplanted in the UBC-STAT3^−/−^ model. In addition, the pharmacologic inhibition of HA synthesis with 4-MU abrogated the ability of IL-10 to induce regenerative wound healing, which suggested an essential role for HA. Of note, this pathway is also consistent with a recent study by Shi and colleagues (47) that showed that IL-10 inhibits fibrosis *in vitro* in hypertrophic scar fibroblasts by facilitating crosstalk between the PI3K/AKT and STAT3 signal transduction pathways. These researchers also showed that treatment with IL-10 can reduce scarring in murine dermal wounds without an apparent effect on the reconstitution of the dermal appendages. However, these wounds received recombinant IL-10 for 7 consecutive days and the underlying mechanisms for improved healing were not completely elucidated (33). Our work highlights the central role IL-10 plays in STAT3-dependent regulation of HA, specifically within the antifibrotic phenotype, which more closely resembles what occurs in the healing of fetal regenerative wounds.

These data strongly implicate AFBs in regenerative healing that is mediated by IL-10, but we do not rule out that other cell types present in the wounded tissue may also play a role in IL-10 responses. For example, other researchers that have worked with models of syngeneic skin grafting have shown that keratinocytes can synthesize IL-10 (48) and activate IL-10–secreting cells (49), thereby playing a key role in the attenuation of inflammation and graft acceptance. IL-10 therapy has been proven to be clinically effective in the treatment of psoriasis by improving keratinocyte-associated pathologic parameters (50), and in the treatment of psoriatic arthritis by affecting endothelial activation, leukocyte recruitment, and effector function (51). We chose to investigate the dermal fibroblast because it is the main effector cell that produces collagen and other ECM components that are important in the pathogenesis of fibrosis and scarring, as opposed to other resident cell populations in the skin (52, 53). Furthermore, the similarities between fibroblasts that are derived from different models of organ fibrosis may denote that the pathway identified in this study is uniquely suited for common therapeutic intervention (54). It has been recently reported that fibroblasts also function as mediators of the effects of IL-10 in the fetal regenerative phenotype, and that FFBs have a functional phenotype that is characteristically different from that of AFBs (24). The present work is in agreement with that study and provides a mechanistic insight into the signaling pathways involved. Of interest, we observed that both fetal and postnatal dermal fibroblasts expressed cell surface IL-10R1 receptors. Blocking IL-10R1 effectively abrogated the effect of IL-10 on STAT3 signal transduction and the differential regulation of HA synthases, HYALs, and HA production in AFBs. Taken together, our *in vitro* data have elucidated a novel intracellular signaling pathway that regulates the metabolism of HA and explains this innovative role for IL-10 beyond its accepted immune-regulatory mechanism.

Our data suggest that IL-10 up-regulates HA by increasing its synthesis and decreasing its degradation. Specifically, we found that IL-10 up-regulates HAS1 and HAS2, which are known to produce high-molecular-weight fetal-type HA, and down-regulates the major HYALs, including the HYAL1, HYAL2, and KIAA1199. KIAA1199 expression is involved in a newer HA degradation pathway that has been suggested to play a greater role in the skin than the pathways that involve HYAL1 or HYAL2 (22). This may lead to the hypothesis that addition of HA is sufficient to achieve regenerative tissue repair; however, we and others have shown that, although the addition of high-molecular-weight HA to wounds restores tissue architecture to more closely resemble that of uninjured dermis, it does not reconstitute the dermal appendages and hair follicles in these wounds (55). This result suggests that IL-10 and IL-10–induced HA may be essential for a complete structural and functional recapitulation of normal architecture after injury.

Although others have previously shown that STAT3 is important in regulating genes that are related to fibrosis (41, 56, 57), the precise molecular mechanisms that mediate this effect remain to be determined, in particular, the cytokine-specific molecular feedback mechanisms of STAT3 activation that are at interplay in driving the profibrotic *vs*. antifibrotic deposition of ECM. This was shown in a recent study by O'Reilly and colleagues (58), who found that even though both IL-6 and IL-10 cytokines use STAT3 downstream of receptor engagement, the ultimate response is different. Whereas IL-6 resulted in increased deposition of collagen-I, IL-10 could not induce gremlin or collagen. Together, these findings also help explain why activation of the STAT3 pathway mediated by IL-10, which results in HA regulation, may be a necessary component for the recapitulation of true regenerative tissue repair, as shown in our studies.

In summary, we have provided evidence for a novel role for IL-10, the regulation of HA, as an essential component of the antifibrotic and regenerative effects of IL-10. The central role that STAT3 plays in the regenerative mechanisms induced by IL-10 is underscored and may have significant clinical and translational applications that would improve the care of patients who cope with debilitating conditions, including pulmonary and renal fibrosis, intra-abdominal adhesions, some traumatic injuries, burn scars, and scleroderma.

## Abbreviations

4-MU: 4-methylumbelliferone
4-OHT: 4-hydroxy tamoxifen
AFB: adult fibroblast
BGS: bovine growth serum
ECM: extracellular matrix
FFB: fetal fibroblast
HA: hyaluronan
HAS: hyaluronan synthase
HMW-HA: high-molecular-weight hyaluronan
HYAL: hyaluronidase
IL-10R: IL-10 receptor
PCM: pericellular matrix
p-STAT3: phosphorylated signal transducer and activator of transcription 3
shRNA: short hairpin RNA
STAT3: signal transducer and activator of transcription 3
TU: transducing unit
UBC: ubiquitin C
